# Supplementary material for: The NICU flora: An effective technique to sample surfaces
Source: PLoS One. 2021 Sep 23;16(9):e0257821. doi: 10.1371/journal.pone.0257821 (PMC8460043; doi:10.1371/journal.pone.0257821)
Supplement: Attachment — Submitted filename: pone review.docx [file pone.0257821.s002.docx]

Thank you for invite me as reviewe, here some review for the manuscript

1. It showed that swab sampling was done before gauze-pad, will it make any differences if you switched the surface sampling order; the gauze-pad first then swab?

2. From a positive environmental culture result, is there a relationship with the condition of the previous patient who occupied the same incubator?

3. Can you tell me about your routine incubator cleaning? Is there a decrease in environmental culture (since the environmental culture is carried out several times with a certain time span)

4. How is the condition of the previous patient with clinical sepsis and positive culture showing HAI, are there new patients who are admitted with symptoms of sepsis?

5. How to inoculate in a culture medium with a gauze sampling method? Won't it be easier to inoculate if you had it done with swab?
